# Different vulnerability of fast and slow cortical oscillations to suppressive effect of spreading depolarization: state-dependent features potentially relevant to pathogenesis of migraine aura

**DOI:** 10.1186/s10194-023-01706-x

**Published:** 2024-01-15

**Authors:** Tatiana M. Medvedeva, Maria P. Smirnova, Irina V. Pavlova, Lyudmila V. Vinogradova

**Affiliations:** 1 Department of Molecular Neurobiology, Institute of Higher Nervous Activity and Neurophysiology, Russian Academy of Sciences, Butlerova Street 5A, 117485 Moscow, Russia; 2 Department of Conditioned Reflexes and Physiology of Emotion, Institute of Higher Nervous Activity and Neurophysiology, Russian Academy of Sciences, Butlerova Street 5A, 117485 Moscow, Russia

**Keywords:** Spreading depolarization, Cortical spreading depression, Migraine, Aura, Animal models

## Abstract

**Background:**

Spreading depolarization (SD), underlying mechanism of migraine aura and potential activator of pain pathways, is known to elicit transient local silencing cortical activity. Sweeping across the cortex, the electrocorticographic depression is supposed to underlie spreading negative symptoms of migraine aura. Main information about the suppressive effect of SD on cortical oscillations was obtained in anesthetized animals while ictal recordings in conscious patients failed to detect EEG depression during migraine aura. Here, we investigate the suppressive effect of SD on spontaneous cortical activity in awake animals and examine whether the anesthesia modifies the SD effect.

**Methods:**

Spectral and spatiotemporal characteristics of spontaneous cortical activity following a single unilateral SD elicited by amygdala pinprick were analyzed in awake freely behaving rats and after induction of urethane anesthesia.

**Results:**

In wakefulness, SD transiently suppressed cortical oscillations in all frequency bands except delta. Slow delta activity did not decline its power during SD and even increased it afterwards; high-frequency gamma oscillations showed the strongest and longest depression under awake conditions. Unexpectedly, gamma power reduced not only during SD invasion the recording cortical sites but also when SD occupied distant subcortical/cortical areas. Contralateral cortex not invaded by SD also showed transient depression of gamma activity in awake animals. Introduction of general anesthesia modified the pattern of SD-induced depression: SD evoked the strongest cessation of slow delta activity, milder suppression of fast oscillations and no distant changes in gamma activity.

**Conclusion:**

Slow and fast cortical oscillations differ in their vulnerability to SD influence, especially in wakefulness. In the conscious brain, SD produces stronger and spatially broader depression of fast cortical oscillations than slow ones. The frequency-specific effects of SD on cortical activity of awake brain may underlie some previously unexplained clinical features of migraine aura.

**Supplementary Information:**

The online version contains supplementary material available at 10.1186/s10194-023-01706-x.

## Background

Migraine aura is a neurological condition that precedes or accompanies the onset of headache in one-third of migraine patients. Aura symptoms include transient sensory (mainly visual) or motor disturbances. Several lines of evidence suggest that spreading depolarization (SD), a self-propagating wave of massive neuroglial depolarization, underlies the aura symptoms and may be a potential activator of downstream pain pathways in migraine with aura patients [1–3].

Intracranial recordings from experimental animals and patients with acute brain injury have revealed two reliable electrographic markers of SD – (1) large-amplitude negative shift of direct current (dc) potential produced by massive near-complete cellular depolarization in the affected tissue and (2) transient depression of ongoing electrical activity [4] resulted from reversible disruption of neuronal signaling [5]. The temporary silence of cortical activity sweeping across the cortex is supposed to underlie spreading negative symptoms of migraine aura [2, 3, 6]. However, surface electroencephalographic (EEG) recordings, in migraine patients failed to demonstrate consistent electrographic abnormalities during migraine attacks [7, 8]. Routine EEG technique is unable to detect dc potential shifts, the gold standard hallmark of cortical SD. The failure to reveal suppression of EEG signal during migraine aura was related with insufficient sensitivity of standard clinical EEG to reveal spatially/temporary restricted depression of cortical activity during SD [2].

Evidence of electrocorticographic (ECoG) depression during SD have been mainly obtained in anesthetized animals or sedated patients with traumatic and ischemic brain injury [4, 5, 9–11]. EEG recordings during migraine attacks are usually performed in conscious humans but experimental studies of SD in awake animals are scarce. Commonly used anesthetics have been shown to impact susceptibility of neuronal tissue to SD [12–14] but their effect on SD-induced ECoG depression have never been studied in details. A role of anesthesia in suppressive effects of SD is usually neglected although general anesthesia is known to change the functional state of cortical tissue and brain network activity that can potentially modify SD effects on spontaneous cortical activity. Some experimental studies did not find significant differences in ECoG effects of SD between awake and anesthetized rodents [15, 16] while others mentioned incomplete (partial) suppression of spontaneous cortical oscillations during SD in awake animals [17, 18].

Migraine aura/SD occurs in the undamaged cortex of migraine patients while most experimental approaches of SD induction include direct mechanical or chemical stimulation of the cortex. To exclude potential confounding effects of direct cortical stimulation on cortical activity [19], we initiated SD extracortically –by a pinprick of the amygdala connected with the cortex by a gray matter bridge allowing slow non-synaptic propagation of SD [18, 20, 21]. SD induced in the amygdala cannot reach the cortex via direct pathway because SD is unable to cross the thick layer of myelinated fibers separating the cortex from subcortical structures. Therefore, SD propagates from the amygdala to the cortex via long (12–15 mm) devious paths subsequently invading the striatum, frontal pole and temporal cortex [22].

The amygdala, playing an important role in pain processing, attracts growing attention due to its potential role in migraine pathogenesis [23–26]. SDs involving subcortical structures (basal ganglia, thalamus, amygdala) are referred as a plausible mechanism for some aura symptoms in migraine with aura patients [3, 6, 14, 27]. In awake rats, cortical SD has been shown to occur in association with thalamic SD [14]. Mice with familial hemiplegic migraine (FHM) mutations exhibit enhanced susceptibility to subcortical SD and facilitated cortico-subcortical propagation of SD [27].

We hypothesized that the influence of SD on cortical activity depends on the vigilance state and some of SD-induced changes may be revealed only in the conscious brain. To test the hypothesis, we studied spectral and spatiotemporal features of ECoG alterations induced by a single unilateral SD in awake freely behaving rats and after introduction of anesthesia. Spontaneous activity of the occipital and frontal cortices was analyzed. Transient dysfunction of the occipital cortex is suggested to underlie visual aura, the most common in migraine patients. Changes in activity of the frontal cortex may be involved in generation of motor and language impairments. Given extensive connections of the frontal cortex with arousal- and pain-modulation subcortical nuclei [28, 29], activity of the cortical region may be important for pain perception and consciousness. Our findings show that in the conscious brain SD elicits depression of cortical activity with characteristics some of which are absent in anesthetized animals and may underlie several clinical symptoms of migraine aura.

## Materials and methods

### Subjects

Adult male Wistar rats (350–450 g, Scientific center for Biomedical Technologies of the Federal Medical and Biological Agency, Russia) were housed in a temperature-controlled vivarium (22˚C ± 2˚C, a 12-h light/dark cycle, lights on at 08.00 h) with food and water ad libitum. All experimental procedures were conducted in accordance with the ARRIVE guidelines and Directive 2010/63/EU for animal experiments. The study protocol was approved by the Ethics Committee of the IHNA RAS (protocol N1 from 01.02.2022). Every effort was made to minimize animal suffering and to ensure reliability of the results.

### Stereotaxic surgery

**Fig. 1:**
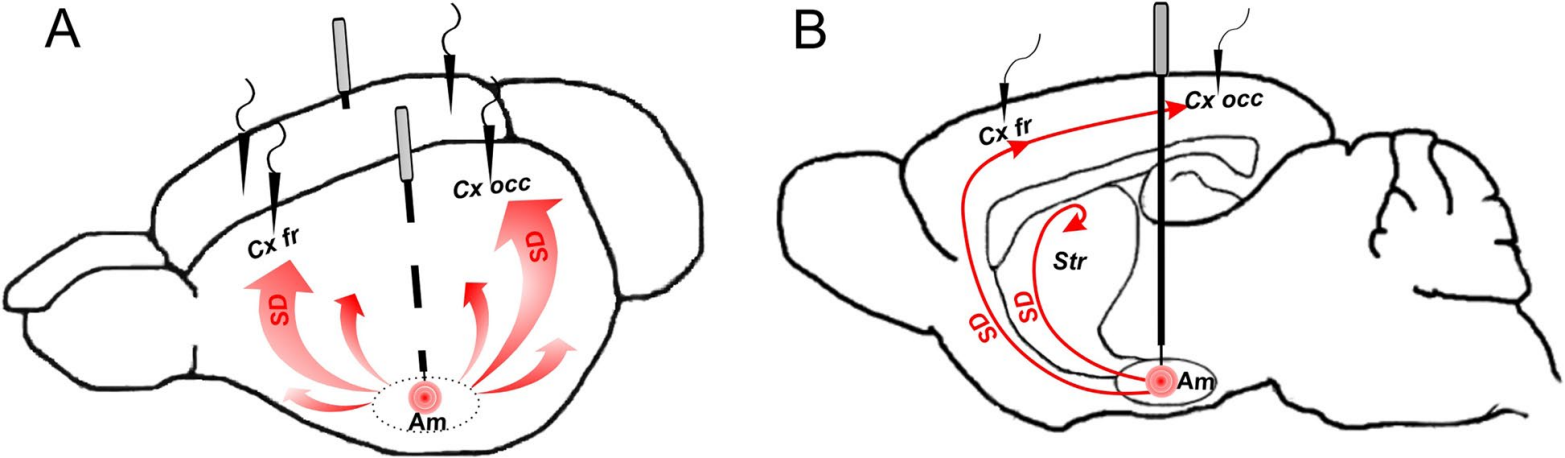
*Pathways of SD propagation from the amygdala to the cortex*. SD was triggered in the amygdala (Am) by its micro-injury via preliminary implanted guide cannula. Cortical activity was recorded in the frontal (Cx fr) and occipital (Cx occ) regions of the cortex using implanted recording electrodes. SD propagated from the injury site to the cortical regions by invading temporal cortex (**A**) and the striatum/frontal pole (**B**)

Under isoflurane anesthesia, rats were bilaterally implanted with electrodes for SD/ECoG recording and guide cannulas for SD induction (Fig. 1). Recording electrodes (insulated silver or nichrome wire, diameter of 0.25–0.30 mm) were positioned in the frontal (AP: + 1.2, ML: ± 2.3 mm, DV:—1.8) and occipital (AP: -5.88, ML: ± 3.5 mm, DV:—1.5 mm) cortices [30]. Reference electrode was placed over the cerebellum. Stainless steel guide cannulas (23 gauge) aimed at the basolateral nuclei of the amygdala (AP: -2.76, ML:—4.8 mm DV:—7.5 mm) of the left and right hemispheres. The guide cannulas, recording electrodes and pin connector were fixed on the skull with acrylic dental plastic. A 30-gauge stylus of the same length as guide cannula was inserted into it to prevent clogging. During three-four days before the start of experiments, all animals were pre-handled and habituated to the stylus removal.

### Initiation of SD and recording of cortical activity

Experiments started two weeks after the surgery.In each rat,three tests with a week interval were performed – the first and second tests under wakefulness and the third test after introduction of urethan anesthesia (1.5 mg/kg, i.p.). In each test, rats were individually placed in a shielded chamber and the implanted connector was attached to the recording cable and spontaneous cortical activity was recorded before (baseline) and after bilateral pinprick of the amygdala as described previously [18, 21]. Briefly, the needle was inserted into the guide cannulaand extended 1.0 mm from its tip, thus providing a small standard damage of the neuronal tissue (Supplementary Fig. S1). As reported previously [21], the local injury of the amygdala triggered SD with about 60% probability. In the present study, we analyzed recordings obtained in rats with histologically verified damaging the basolateral amygdala (BLA) that exhibited maximal susceptibility to SD [20, 21]. At a week interval, the BLA pinprick induced SD with similar probability in test 1 (59%, 19/32) and test 2 (72%, 23/32) in awake rats (*p* = 0.43, Fisher exact test) and reduced probability (19%, 6/32 in test 3) in anesthetized rats (*p* < 0.001). Due to probabilistic nature of SD occurrence after the amygdala damage, simultaneous bilateral microinjury of the amygdala produced three outcomes—a single bilateral SD, a single unilateral SD or no SD. Most rats (14/16) exhibited variable response in three repeated tests (bilateral/unilateral/no SD). In the present study, only artefact-free LPF recordings with induction of by a unilateral SD were analyzed. Tests with initiation of a bilateral SD and with lesions localized outside the BLA were excluded from analysis. Recordings from tests, in which the bilateral BLA pinprick failed to trigger SD, were used as sham controls.

Full-band cortical activity (0–100 Hz, 1 kHz sampling rate) was recorded using a four-channel, high-input impedance (1 gΩ) dc amplifier and a/d converter (E14-440, L-Card, Russia) with simultaneous video-monitoring of behavior. Cortical activity was recorded during 15-min before (baseline activity) and 15 min after the amygdala stimulation. In off-line analysis of cortical activity, recordings of local field potential (LFP) were filtered with bandpass filters 0–50 Hz (direct current, dc) and 1–50 Hz (ECoG). SD was identified by the occurrence of a high-amplitude dc potential shift, the most reliable electrophysiological manifestation of SD.

### Data processing

For spectral analysis, artifact-free 600-s epochs of LFP recordings of baseline activity and after induction of a single unilateral SD (*n* = 13) or no SD (sham stimulation, *n* = 6) were used. The segments were filtered with a highpass (1 Hz highcut) and bandstop (48 Hz lowcut and 52 Hz highcut) Butterworth digital filters using *scipy* package (all calculations here and below were performed in Python language). Further, the 600-s epochs were divided into 10-s length intervals and the mean power for each interval in each frequency band was evaluated without overlapping using *fft*function from *numpy*package. Spectral power was computed using a Fast Fourier Transform (FFT) routine for five frequency bands: delta (1–4 Hz), theta (4–8 Hz), alpha (8–12 Hz), beta (12–25 Hz) and gamma (25–50 Hz). The amplitude of ECoG depression during SD was measured as a percentage of average power during depolarization phase of SD relative to the baseline level. Data processing was performed by T.M. blinded to SD presence during the analyzed period. Spectrograms were obtained using *specgram* function from *matplotlib* package with 2048 data points (approximately 2 s) used in each block for the FFT and overlapping of 90%.

### Histology

For histological verification of amygdala injury and localization of recording electrodes, animals were euthanized and perfused intracardially with 0.9% saline after the end of the experiments. The brains were removed, stored in 10% formalin for 48 h, sectioned in coronal 50-μm slices and stained with 0.1% cresyl violet.

### Statistical analysis

Statistical analysis was performed using Statistica software12.0 (StatSoft). Significant difference in spectral power dynamics between baseline and post-SD periods was assessed using ANOVA for repeated measures with SD as a between-subject factor and time (10-s intervals) as a within-subject factor. One-way ANOVA was used for post-hoc comparison of spectral power dynamics during baseline and post-SD periods. Inter-regional differences in the ECoG power magnitudes were estimated with Wilcoxon signed-rank test. The ECoG power magnitudes in awake and anesthetized animals were compared using Mann–Whitney test. Fisher exact test was used to compare behavioral changes in rats with SD and sham-treated animals. The data were expressed as mean ± S.E.M. The significance was set at *p* < 0.05.

## Results

### Propagation of SD to the cortex and SD-induced ECoG depression in awake conditions and after introduction of anesthesia

Local microinjury of the amygdala triggered a single SD wave that non-synaptically propagated to the frontal and occipital regions of the cortex via gray matter bridges connecting the amygdala and cortex (Fig. 1) [18, 20, 22]. SD induced in the amygdala can reach the cortical regions via the piriform cortex [20] (Fig. 1A) and through the striatum and frontal pole (Fig. 1B). Amygdalar SD always spreads to the striatum [18] and expires at the boundaries with corpus callosum. But, as shown previously [22], SD can leave the striatum and penetrate the frontal cortex via a rostral pathway. By sequential invading adjacent temporal lobe and striatum, SD reached the frontal and occipital cortices in about three min post-injury, irrespective of the vigilance state (Table 1).

**Table 1:** Parameters of dc potential shifts associated with SD in awake and anesthetized rats

*Cortical region*	*SD latency (s)*	*SD duration (s)*	*SD amplitude (mV)*
**Awake rats (*n* = 7)**			
Frontal cortex	169 ± 7	45 ± 2	8.0 ± 0.7
Occipital cortex	160 ± 5	41 ± 2	6.1 ± 0.7
**Anesthetized rats (*n* = 6)**			
Frontal cortex	200 ± 5*	63 ± 3*	8.7 ± 0.3
Occipital cortex	220 ± 20*	57 ± 3*	5.8 ± 1.2

^*^-significant difference between awake and anesthetized rats (*p* < 0.05, Mann–Whitney test)

During the first two minutes, i.e. before arrival to the frontal and occipital cortices, SD traveled over deep brain regions, including the striatum. When SD invaded the striatum (40–100 s after the amygdala pinprick), rats exhibited several episodes of forced circling, a reliable behavioral marker of striatal SD [18]. Introduction of anesthesia slightly increased the latencies of SD appearance in the cortex and durations of SD-associated dc-potential shifts (Table 1).

**Fig. 2:**
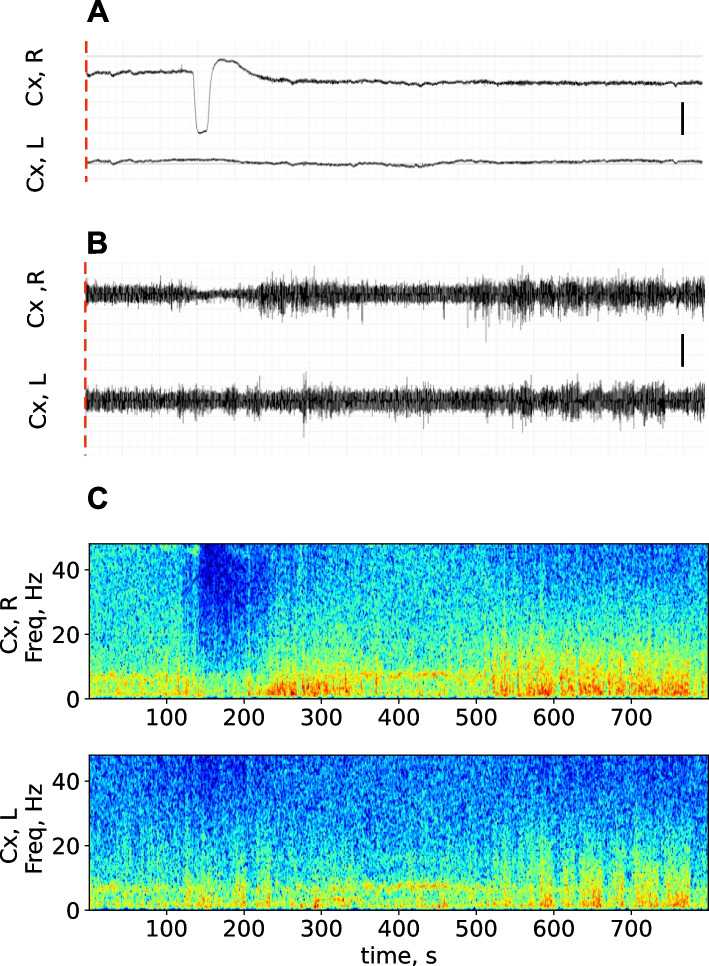
*Depression of spontaneous cortical activity induced by unilateral SD in awake rats.* Typical recordings of dc potential (**A**), filtered ECoG (**B**) and spectrogram (**C**) of the 800-s fragment obtained in homotopic sites of the right (Cx, R) and left (Cx, L) occipital cortex of the two hemispheres immediately after a focal microinjury of the right amygdala (marked by red dashed line at the onset of recordings). Calibration bars – 2 mV (**A**) and 0.2 mV (**B**). The time scale is the same in A, B, C and shown below the spectrogram. A single SD event (dc shift) appeared in the right occipital cortex in 150 s after its initiation in the amygdala (**A**) and induced mild suppression of ipsilateral ECoG amplitude (**B**, **C**)

**Fig. 3:**
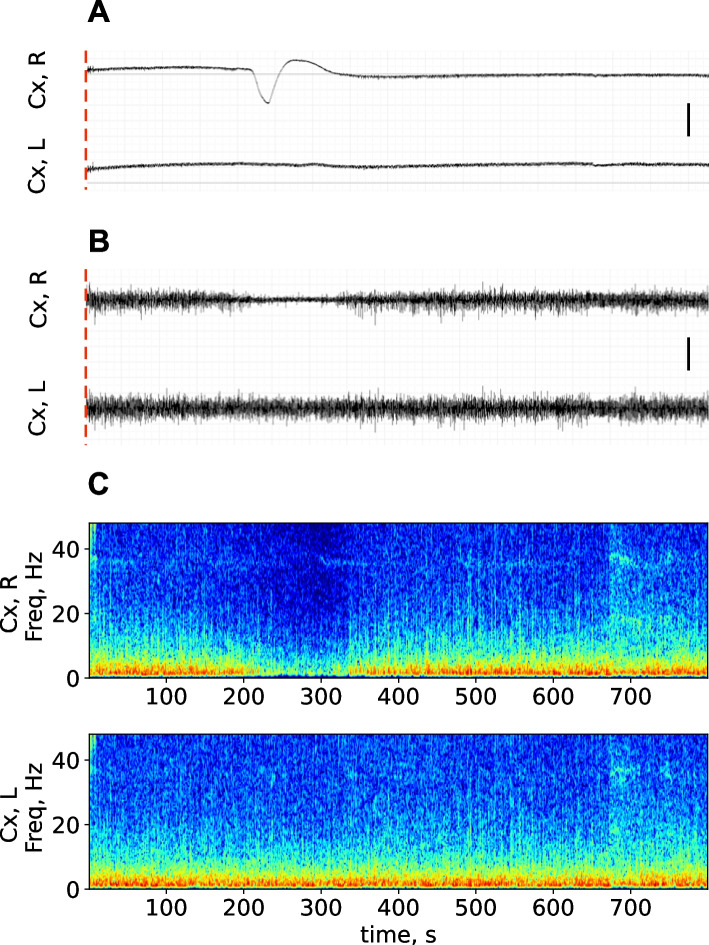
*Depression of spontaneous cortical activity induced by unilateral SD in urethane-anesthetized rats.* Typical recordings of dc potential (**A**), filtered ECoG (**B**) and spectrogram (**C**) of the 800-s fragment obtained in homotopic sites of the right (Cx, R) and left (Cx, L) occipital cortex of the two hemispheres immediately after a focal microinjury of the right amygdala (marked by red dashed line at the onset of recordings) in the rat shown in Fig. 2 under awake conditions. Calibration bars – 2 mV (**A**) and 0.2 mV (**B**). The time scale is the same in A, B, C and shown below the spectrogram. A single SD event (dc shift) appeared in the right occipital cortex in 220 s after its initiation in the amygdala (**A**) and induced pronounced ECoG flattering in the ipsilateral cortex (**B**, **C**)

As mentioned above, here we analyzed effects of a unilateral SD induced by a bilateral BLA pinprick. Tests, in which the damage failed to trigger SD, were used as sham controls. Electrographic manifestations of a single unilateral SD arrived to the occipital cortex recorded in the same rat under awake and anesthetized conditions are shown on Figs. 2 and 3, respectively (the traces were obtained immediately after the BLA microinjury).

In awake conditions, SD appeared in the cortex in 150 s after its initiation in the amygdala (Fig. 2A). Visual inspection of the ECoG recording and spectrogram showed that SD transiently reduced amplitude of ipsilateral cortical activity without changes in the contralateral cortex (Fig. 2B, C).

Under urethan anesthesia, SD appeared in the cortex a bit later—at 220 s post-injury (Fig. 3A) and produced pronounced ipsilateral ECoG depression (Fig. 3B, C) that corresponded well to the pattern previously described in anesthetized animals [4, 5, 10].

### Effects of SD on spectral and spatiotemporal characteristics of cortical oscillations in awake conditions and after induction of anesthesia

**Fig. 4:**
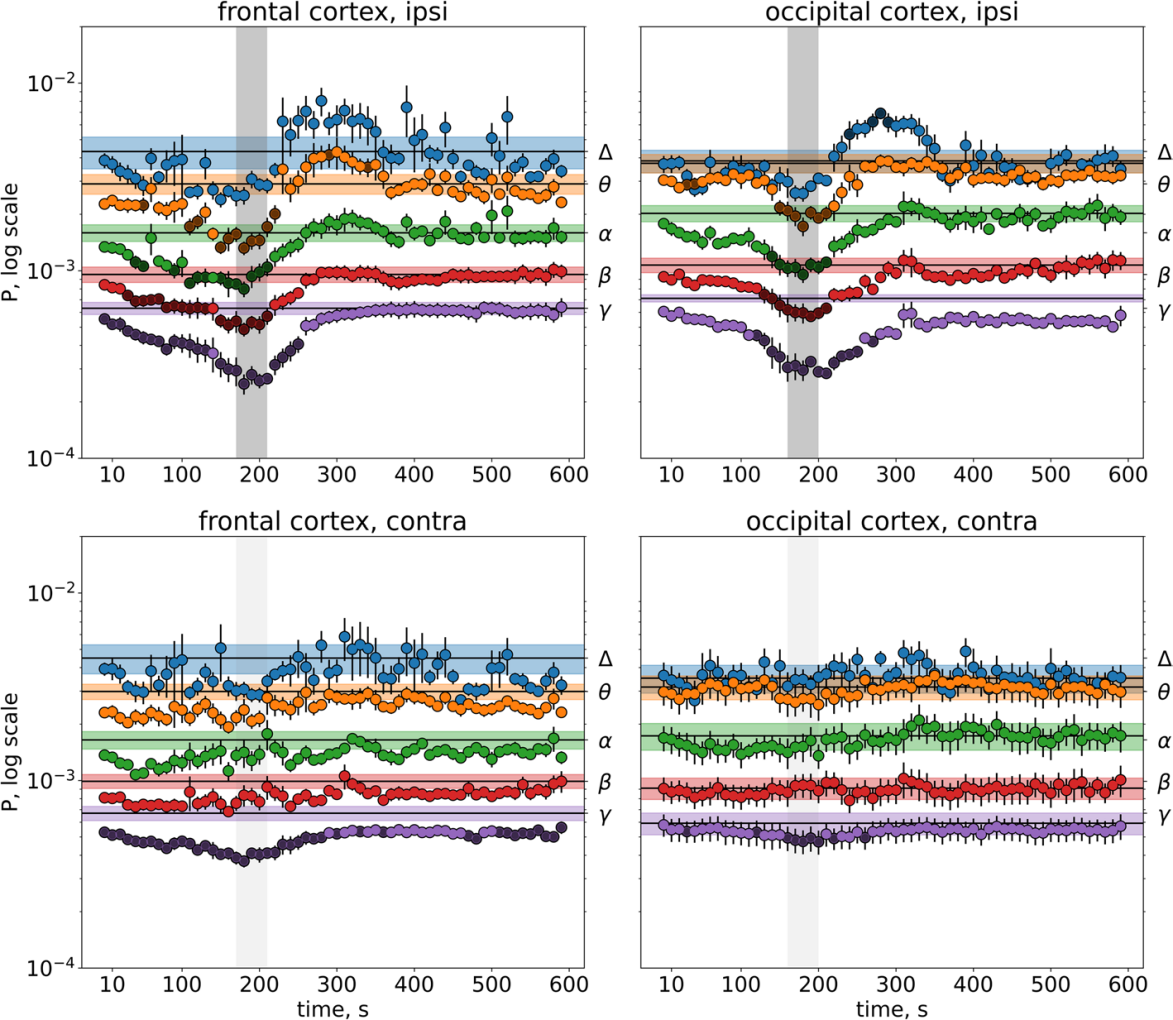
*Effect of unilateral SD on ECoG power in different frequency bands in awake rats*. Graphs show mean power of delta (1–4 Hz), theta (4–8 Hz), alpha (8–12 Hz), beta (12–25 Hz) and gamma (25–50 Hz) oscillations (marked on the right Y-axis) in the frontal (left fragments) and occipital (right fragments) cortices ipsilateral (upper fragments) and contralateral (lower fragments) to SD (*n* = 7). Within each band, lines with shadows mark baseline activity power, circles mark power for 10-s intervals following SD initiation, dark circles indicate intervals significantly differed from the baseline level (*p* < 0.05, one-way ANOVA). Gray vertical areas in the fragments show periods of DC potential shift (depolarization phase of SD) in respective cortical regions

**Fig. 5:**
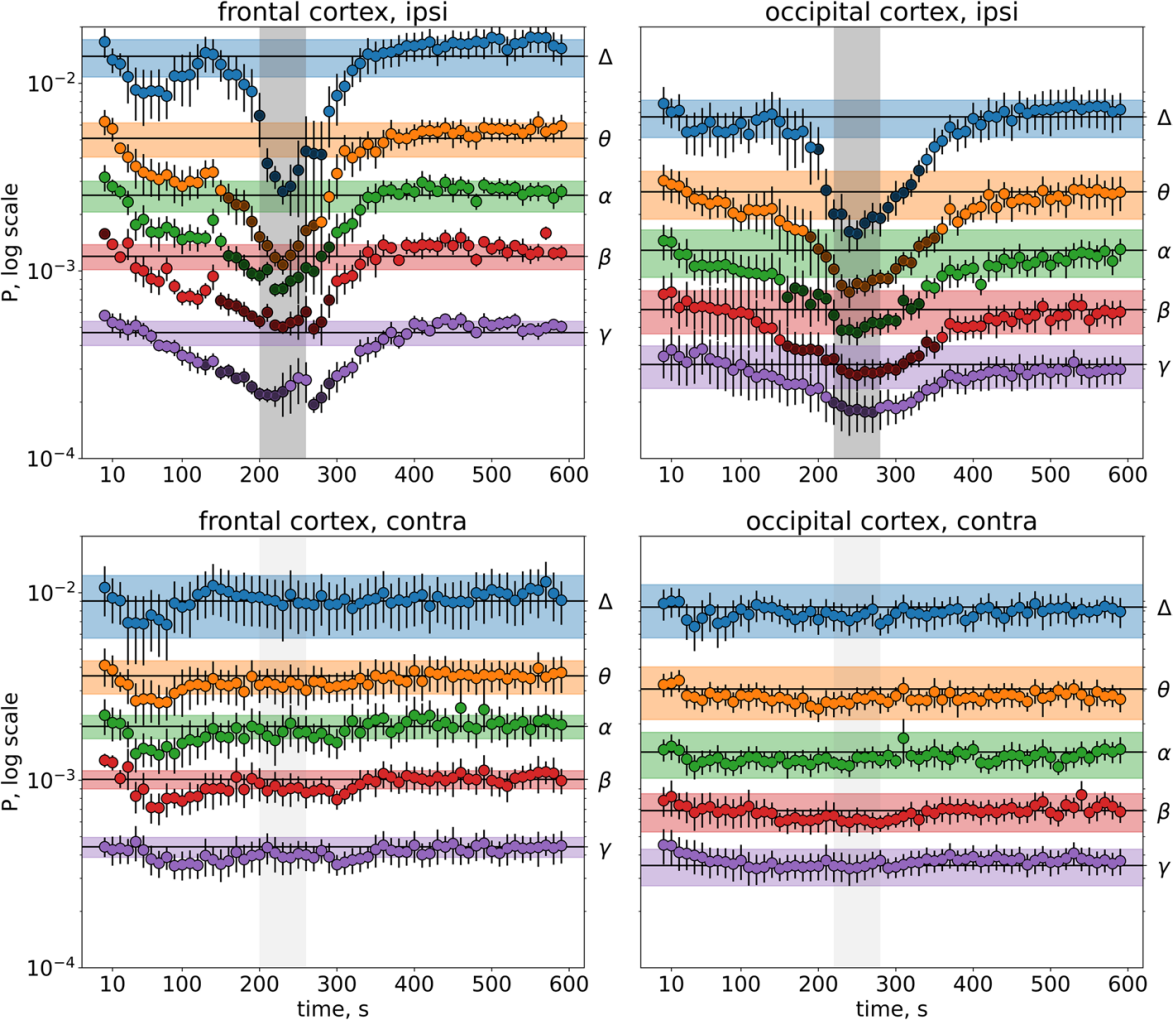
*Effect of unilateral SD on ECoG power in different frequency bands in anesthetized rats*. Graphs show mean power of delta (1–4 Hz), theta (4–8 Hz), alpha (8–12 Hz), beta (12–25 Hz) and gamma (25–50 Hz) oscillations (marked on the right Y-axis) in the frontal (left fragments) and occipital (right fragments) cortices ipsilateral (upper fragments) and contralateral (lower fragments) to SD (*n* = 6). Within each band, lines with shadows mark baseline activity power, circles mark power for 10-s intervals following SD initiation, dark circles indicate intervals significantly differed from the baseline level (*p* < 0.05, one-way ANOVA). Gray vertical areas in the fragments show periods of DC potential shift (depolarization phase of SD) in respective cortical regions

Arrival of SD to the cortex produced a pronounced drop of cortical activity power (Figs. 4 and 5). In the ipsilateral cortex, significant effects of SD on dynamics of oscillation power were found across all frequency bands in both awake and anesthetized conditions (*p* < 0.001, Table S1). In the unaffected contralateral cortex, SD significantly affected the gamma oscillation power only in awake rats (*p* < 0.001, Fig. 4, Table S1). Sham stimulation, i.e. identical amygdala damage without SD induction, did not change cortical activity (Supplementary Fig. S2), indicating that the ECoG depression was produced by injury-induced SD but not the injury per se.

As seen in Figs. 4 and 5, the power drop peaked during the depolarization phase of SD (marked by gray vertical areas). Under awake condition, slow delta (1–4 Hz) oscillations did not reduce their power during SD and even overshot afterwards in the occipital cortex (Fig. 4). On contrary, fast gamma (25–50 Hz) activity showed the pronounced depression, especially in the frontal cortex where it started long before SD arrival (Fig. 4).

After introduction of anesthesia, the early pre-SD depression disappeared and gamma power decline started near the onset of dc potential shift while depression of other frequency band oscillations began to outlast termination of dc-shift, especially in the occipital cortex (Fig. 5).

**Fig. 6:**
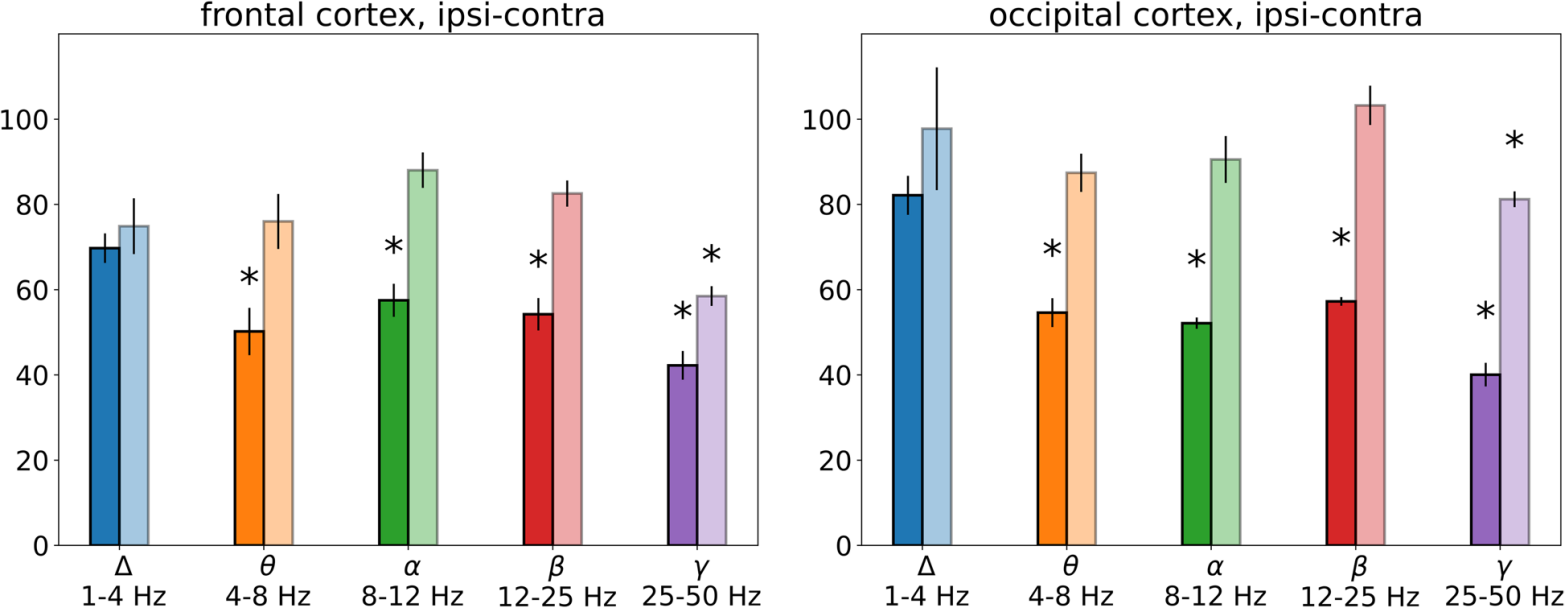
*Magnitudes of SD-induced ECoG depression in different frequency bands in awake rats.* The bars represent percentages of average power of cortical oscillations for each frequency range in the ipsilateral (dark bars) and contralateral (light bars) regions during depolarization phase of SD (dc-shift) relative to the baseline. *—*p* < 0.05 – significant difference from the respective baseline level within each frequency band

**Fig. 7:**
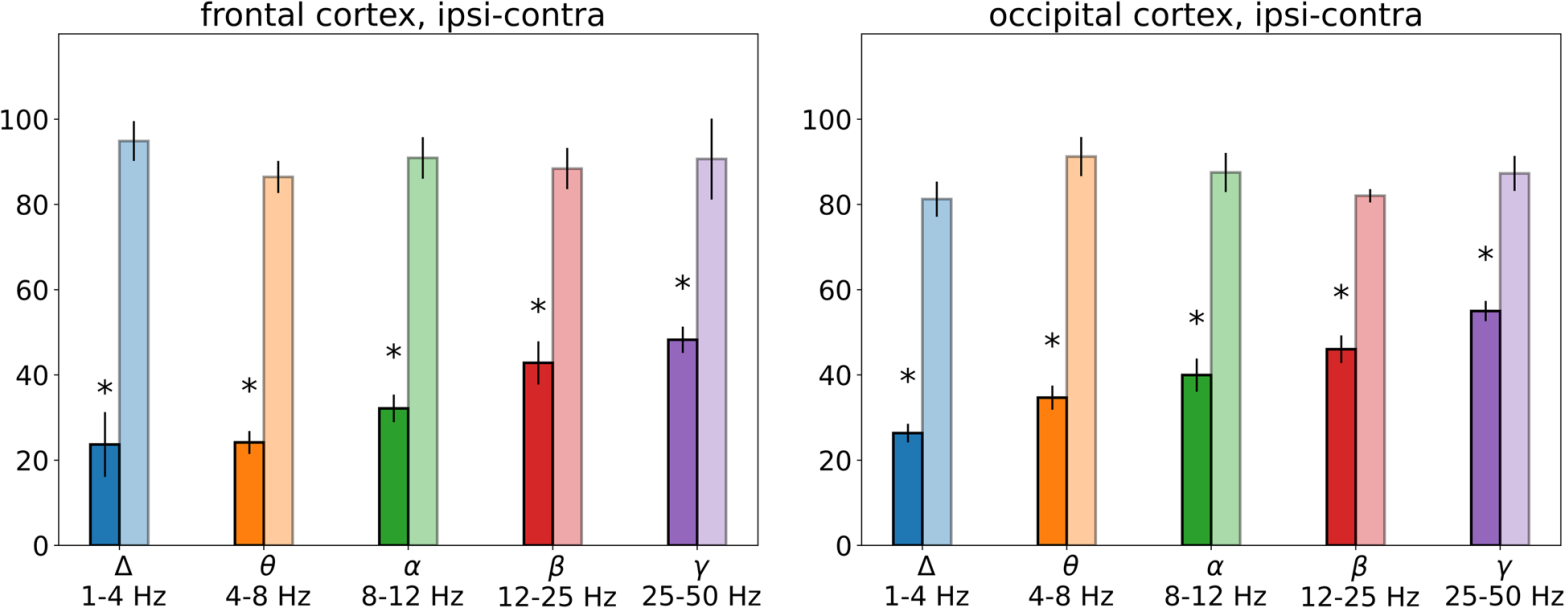
*Magnitudes of SD-induced ECoG depression in different frequency bands in anesthetized rats*. The bars represent percentages of average power of cortical oscillations for each frequency range in the ipsilateral (dark bars) and contralateral (light bars) regions during depolarization phase of SD (dc-shift) relative to the baseline. *—*p* < 0.05 – significant difference from respective baseline levels within each frequency band

The degrees of the SD-induced depression in different frequency bands that were expressed as percentages of average power during dc potential shift relative to respective baseline levels within each band are compared in Figs. 6 and 7. Under awake condition (Fig. 6), power of delta oscillations did not change significantly, theta (4–8 Hz), alpha (8–12 Hz) and beta (12–25 Hz) oscillation power showed two-fold reduction in the ipsilateral cortex (*p* < 0.05, Wilcoxon test), high-frequency gamma (25–50 Hz) activity exhibited maximal decrease and involved both ipsi- and contralateral cortices (to about 40% and 60% of baseline level, respectively).

After introduction of anesthesia, SD elicited wideband depression of ipsilateral cortical activity (*p* < 0.05, Wilcoxon test, Fig. 7). In anesthetized animals, the strongest (4–fivefold) reduction was found in the slow delta range (to a mean 20% of baseline), faster cortical rhythms were less depressed and minimal changes were observed in the high-frequency gamma band (to a mean 50% of baseline). Comparison of awake and anesthetized rats showed that under wakefulness SD produced significantly weaker suppression of delta, theta, alpha and beta oscillations but stronger depression of fast gamma activity than in anesthetized rats (*p < 0.05, *Fig. 6*).*

Although maximal drop of cortical activity power was time-locked the depolarization phase of SD, ECoG depression usually lasted two threefold longer (Table 2, Figs, 4 and 5). In wakefulness,the longest suppression was observed in high-frequency gamma (25–50 Hz) range—up to 250 s, i.e. four–sixfold longer than dc shift. Anesthesia shortened the gamma depression but lengthened silencing cortical oscillations in other frequency bands due to slow post-SD recovery, especially in the occipital cortex.

**Table 2:** Duration of depolarization phase of SD (DC shift) and SD-induced depression of ipsilateral cortical oscillations in different frequency bands in awake and anesthetized rats

	**Frontal cortex**	**Occipital cortex**
**Awake rats** (*n* = 7)		
DC shift (s)	40	40
Delta (1–4 Hz) (s)	-	-
Theta (4–8 Hz) (s)	110	90
Alpha (8–12 Hz) (s)	130	80
Beta (12–25 Hz) (s)	180	90
Gamma (25–50 Hz) (s)	250	150
**Anesthetized rats** (*n* = 6)		
DC shift (s)	60	60
Delta (1–4 Hz) (s)	90	140
Theta (4–8 Hz) (s)	120	170
Alpha (8–12 Hz) (s)	140	180
Beta (12–25 Hz) (s)	150	200
Gamma (25–50 Hz) (s)	130	60

Thus, slow and fast cortical oscillations exhibited pronounced difference in vulnerability to suppressive effect of SD that strongly depended on the vigilance state. Slow delta oscillations were not depressed by SD and even aggravated afterwards in awake rats but were maximally reduced during SD in anesthetized animals. On contrary, fast gamma activity showed the strongest and longest power decline during SD in awake animals but was minimally affected by SD in anesthetized conditions.

### Remote suppressive effects of SD on fast cortical oscillations in awake state

As mentioned above, unilateral SD induced in awake rats exerted a significant effect on contralateral gamma oscillations (Table 1) eliciting bilateral gamma depression (Figs. 4 and 6). In both frontal and occipital regions of the unaffected contralateral cortex, power of gamma oscillations showed significant, though milder, decline similar to that observed in the ipsilateral to SD cortex—brief drop in the occipital cortex and prolonged building-up suppression in the frontal cortex.

Gamma power in the frontal cortex started to decline immediately after induction of SD in the distant subcortical region, progressively dropped till SD arrival to the recording cortical site, peaked during dc shift and recovered with its termination. Alpha and beta bands also showed early-onset depression but shorter than gamma one and only in the ipsilateral frontal cortex. During the early period of the depressed fast cortical activity (first two minutes post-injury), SD propagated over the remote subcortical sites (amygdala, striatum) and distant cortical (prefrontal and temporal) regions as shown in Fig. 1 and described previously [18, 20–22].

**Fig. 8:**
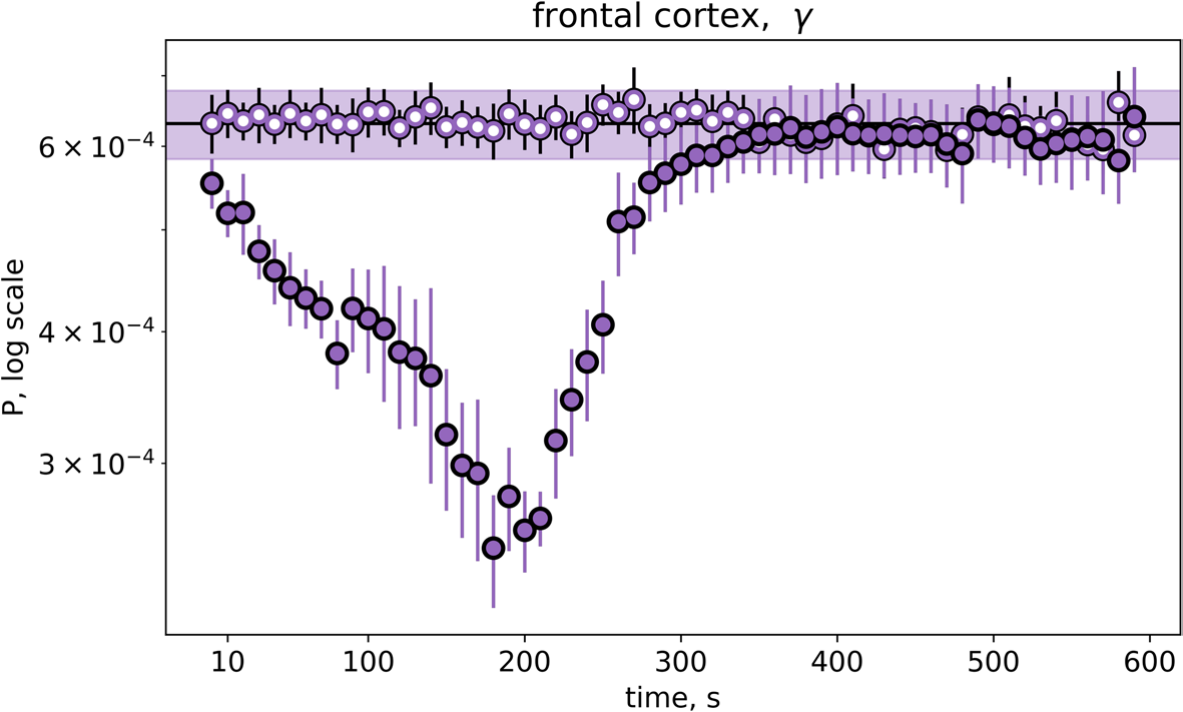
*Frontal gamma oscillations are depressed only after injury triggering SD*. Mean power of gamma activity recorded in the ipsilateral frontal cortex of awake rats following amygdala stimulation induced (dark circles, *n* = 7) and not induced (white circles, sham injury, *n* = 6) SD. The baseline level is marked by line with shadow. Only stimulation triggering SD elicited transient reduction of frontal gamma power

To clarify whether the early frontal gamma depression was produced by mechanical stimulation per se or by SD triggered by the stimulation, we compared changes in frontal gamma power following amygdala pinprick that induced a single unilateral SD and sham stimulation that failed to initiate SD (Fig. 8). As can be seen, sham stimulation did not change the power of gamma oscillations while identical stimulation triggering SD elicited strong gamma depression. Thus, the suppression of high-frequency gamma oscillations preceding SD arrival to the cortex of awake rats is likely to reflect remote effects of SD traveling over the distant subcortical and cortical regions.

### Behavior during propagation of SD from the amygdala to the cortex

To identify behavioral patterns associated with SD, we compared data of video-monitoring obtained after BLA pinpricks induced no SD (sham-treated animals) or a unilateral SD. During the 15-min observation period, rats of both sham and SD groups exhibited exploratory behavior with sniffing and rearing, periods of grooming, quiet standing and lying. All rats with SD (7/7) showed forced circling when SD invaded the striatum (40–100 s) and recurrent episodes of freezing that started to appear immediately after the BLA pinprick and repeatedly occurred during subsequent SD propagation from the amygdala to the cortex (4–5 min). Most animals with SD (6/7) also exhibited repeated bouts of purposeless masticatory jaw movements during SD traveling (1- 4 min after pinprick) and head shakes/wet dog shakes following grooming behavior during late post-SD period (5–15 min). In sham group (*n* = 6), rats never expressed circling behavior, two animals showed several episodes of freezing and masticatory movements and one rat exhibited wet dog shakes after late grooming. As compared to sham-treated animals, rats with SD more frequently expressed circling behavior (*p* < 0.005), episodes of freezing (*p* < 0.05), mastication (*p* < 0.05) and head/wet dog shakes (*p *< 0.05, Fisher test).

## Discussion

### Suppressive effect of SD on spontaneous cortical activity of awake and anesthetized animals

The present study shows that vulnerability of slow and fast cortical oscillations to suppressive effects of SD profoundly differs, especially in awake state. In freely behaving rats, SD is accompanied by strong cessation of fast cortical oscillations, particularly pronounced in the gamma (25–50 Hz) range, while the slowest (delta, 1–4 Hz) activity was not depressed during SD but even increases afterwards. The pattern of partial ECoG depression may explain incomplete cessation of cortical activity during SD previously reported in awake rats and rabbits [17, 18] and the failure of most clinical studies to detect clear EEG depression during migraine aura in conscious patients [7, 8]. Similar to our experimental findings, MEG/EEG data from migraine patients reported (1) ipsilateral suppression of high-frequency (alpha and gamma) cortical activity during visual aura that was suggested to contribute to inhibition of visual function and phosphene generation [31] and (2) increased delta power in the occipital cortex (posterior slow waves) during typical migraine aura [32] and FHM aura [33].

Introduction of anesthesia, despite its rather mild effect on parameters of dc shifts associated with SD, significantly modifies the pattern of SD-induced ECoG depression, weakening suppression of gamma oscillations and intensifying depression of cortical activity in other frequency bands. Under anesthesia, SD is accompanied by wideband suppression of cortical activity with the strongest power drop of slow delta activity and milder reduction of faster oscillations. The result is in line with clinical data obtained in sedated patients with traumatic brain injury in which EEG/ECoG suppression during SD was mainly determined by suppression of slow cortical activity in the delta frequency band (reduction to 47%) while high-frequency oscillations were less depressed [9]. Intense wideband depression found during SD under anesthesia in our study corresponds well to SD-induced complete ECoG depression previously described in anesthetized rabbits, rats, mice and pigs [4, 5, 10].

In both awake and anesthetized states, drop of ipsilateral cortical oscillations power always peaks during DC-shifts confirming the well-known idea that the most prominent deactivation of the cortex occurs during depolarization phase of SD as a result of depolarization block of neuronal activity [4]. However, multiple preclinical evidence show that SD-induced suppression of spontaneous cortical activity lasts significantly longer than the depolarization phase of SD ([e.g., [5]). Our results are in line with the well-known data and show that duration of the ECoG depression may depend on vigilance state, cortical region and type of cortical oscillations.

### Remote effects of SD on high-frequency cortical activity of awake brain

The striking feature of SD effects on cortical activity of awake animals was bilateral depression of high-frequency gamma oscillations induced by unilateral SD. A decrease in gamma power was observed both in the cortex invaded by SD and in the unaffected contralateral cortex with region-specific time courses – short-lasting depression in the occipital cortex and long-lasting early-onset decline in the frontal cortex. Bilateral suppression of alpha band (8–11 Hz) cortical oscillations was described following KCl-induced unilateral cortical SD that was referred as a diaschisis manifestation [34]. Our study shows that the SD diaschisis selectively involves fast cortical oscillations and exhibits state- and region-dependent features. Recently, we reported that in awake rats a single unilateral cortical SD elicited a transient loss of interhemispheric functional interactions, especially pronounced in the beta-gamma frequency bands [35]. The functional decoupling may underlie the ECoG depression produced by contralateral SD.

In the frontal cortex of awake rats, beta and gamma band power began to reduce soon after SD initiation in the BLA and progressively diminished with SD approach to the cortex (during the first two minutes post-injury). Similar depression of beta cortical activity starting long before SD appearance at the recording sites was reported in patients with traumatic brain injury, in which SD occurrence was closely associated with reduced beta band power [36]. Given that the gamma depression preceding cortical SD was absent after sham stimulation not triggering SD and that during the early period SD traveled over the remote subcortical and cortical regions [18, 20–22] (see Fig. 1), we conclude that the early-onset cessation of fast activity was produced by network effects of SD invading distant brain regions. Previously, it has been shown that neuronal (unit) activity and sensory evoked responses of the cerebral cortex were reduced during subcortical (striatal and thalamic) SDs [37, 38]. That is, subcortical SD can alter cortical function by transient elimination of afferent inputs to the cortex and functional disconnection of the cortex from deep brain regions during the depolarization phase of subcortical SD [6, 16]. However, recent experimental evidence indicate that distant effects of SD may be more complex. In awake mice, cortical SD has been reported to elicit transient neuronal activation of the ipsilateral thalamus [39].

The frequency-, state- and region-specific character of the remote effects of SD may explain why it usually remained unnoticed in experimental studies. Also, in most studies SD was initiated within the rodent cortex, the small size of which hampered detecting the distant effects of SD. A role of initiation site localization (the parieto-occipital cortex in most studies and the amygdala in the present work) cannot be excluded. Our experimental design with initiation of SD in remote extracortical region and a significant time lag between SD induction and its arrival to recording points mimics better SD traveling over long distances such as those observed in the human cortex.

It remains unclear why the remote effects of SD are strongly expressed by the frontal cortex. In the cortical region, gamma-band depression preceded SD arrival and involved both affected and unaffected hemispheres (Fig. 4). Urethan anesthesia abolished the early pre-SD and contralateral gamma depression (Fig. 5). Similarly, thalamic activation during cortical SD was eliminated by anesthesia [39]. Anesthetics are known to diminish activity of brainstem arousal nuclei and affect bidirectional communication across the brainstem, thalamus, and cortex. The frontal cortex receiving robust ascending projections from arousal- and pain-modulation brainstem nuclei [28, 29] may be particularly sensitive to the changes in cortico-subcortical interactions. Also, the vulnerability of the frontal cortex may result from its contiguity to subcortical pathways of SD traveling from the amygdala (Fig. 1) that implies the existence of spatial limits for the remote effect expression. At last, anatomical/functional connections between sites driving the remote SD effects and the two cortical regions may differ. The frontal cortex is the most important recipient of a direct input from the periaqueductal gray matter (PAG) while occipital cortex receives only a minor PAG projection [28].

Migraine is a disorder of cortico-subcortical interactions. It is thought that activation of subcortical structures drives symptomatology of premonitory and headache phases of the migraine attack while cortico-thalamic events are accepted to determine sensory manifestations of the aura phase [1, 2, 14, 26]. Cortical SD has shown to invade the visual domain of thalamic reticular nucleus [14] and to activate thalamic ventral posteromedial nucleus [37], which are both relevant to sensory information processing. Aberrant activity of brainstem arousal and nociceptive networks during premonitory period is suggested to initiate migraine attacks [1, 2, 26]. Hyperexcitation of ascending subcortico-cortical pathways can trigger cortical SD in awake animals [40, 41]. On the other hand, SD involving subcortical structures is also referred as a plausible mechanism for some aura symptoms in patients [3, 6, 14].

The present study shows that in awake conditions SD exerts remote effects on fast activity of the cortex and this effect is abolished by urethan anesthesia. This suggests that SD occurring in the conscious brain of migraine patients exerts not only direct local ECoG depression in the affected tissue but may also produce indirect suppression of high-frequency gamma oscillations in distant brain regions.

### Effect of a single unilateral SD induced in the amygdala on spontaneous behavior

The present study shows that traveling SD from BLA to the cortex is reliably accompanies by episodes of forced circling, freezing behavior and “chewing” movements. As shown previously, circling behavior time-locks SD invasion of the striatum [18]. Its reproducible occurrence soon after the BLA pinprick indicates regular propagation of SD initiated in the amygdala to the striatum. Association of cortical SD with freezing behavior has been reported previously [14, 16, 18]. It has been suggested that mechanisms of the SD-related freezing involve the amygdala playing the critical role in expression of the fear and anxiety behavior [16]. Our findings support the idea and show that recurrent episodes of freezing appear immediately after SD initiation in the amygdala. In the present study, new behavioral pattern associated with SD – recurrent masticatory jaw movements – was identified. Given that the behavior is generated by trigeminal circuits controlling orofacial motor function [42], the SD-associated “chewing” movements may indicate activation of downstream nociceptive pathways during SD propagation in the brain.

### Relevance to pathogenesis of migraine aura

Migraine aura is a neurologic condition characterized by transient visual, somatosensory and language symptoms that develop before headache phase of migraine attack. Cortical SD induced in experimental animals represents a highly translational model of the acute neurological deficit. Though experimental SD recapitulates many characteristics of migraine aura in human subjects, some features of SD do not match well clinical pattern of aura [3]. Our study shows that the mismatch may be related, at least partially, to the fact that the main body of our knowledge about electrographic characteristics of SD has been obtained in anesthetized animals. Here, we found that SD elicits more complex changes in cortical activity in the awake state compared to those observed under anesthesia. Some of the changes detected only under awake condition may underlie several unexplained features of migraine aura.

First, *bilateral* aura symptoms are frequently observed in migraine patients but mechanisms of the aura pattern remain unclear based on properties of unilateral SD described in anesthetized animals (depression is confined to the cortex affected by SD). Multiple experimental studies, including the present one, showed that under anesthesia suppressive effect of unilateral SD is confined to the ipsilateral cortex. Here, we found for the first time that in awake conditions the contralateral cortex unaffected by SD also shows transient depression of cortical gamma oscillations. It is known that high-frequency cortical activity plays the critical role in processing of sensory information and impaired regulation of the activity is referred as a hallmark of neurological dysfunction. The ability of unilateral SD to produce in awake brain reversible bilateral depression of gamma oscillations may potentially underlie bilateral sensory disturbances during migraine aura.

Second, visual and somatosensory aura symptoms can appear in rapid succession or simultaneously. Such symptomatology cannot be explained by direct traveling SD over the human cortex due to a long distance between the visual and somatosensory cortical regions. Moreover, functional imaging studies did not find such propagation patterns in patients and showed that the event underlying visual aura propagates along a single gyrus or sulcus [43]. Based on the clinical data, multifocal triggering cortical SD during aura has been suggested [2]. Our study revealed that in wakefulness beta-gamma depression spreads beyond a spatially limited SD event and produces ECoG depression in broader cortical areas not invading them. Restricted traveling SD along the gyrus/sulcus thus can drive visual aura and exert distant effect on activity of the somatosensory cortex, yielding several sensory symptoms simultaneously. Given an important role of high-frequency gamma oscillations in the frontal cortex in network-level computations, their prolonged depression produced by SD in awake brain may underlie cognitive impairments during migraine attacks.

Finally, the majority of migraine patients exhibit positive sensory symptoms which remain unexplained based on mainly suppressive effect of SD on cortical activity. Previously, we have shown that in awake rats cortical SD is followed by transient hyperexcitation of the ipsilateral cortex [35]. The present study confirmed the finding and showed that in awake state SD is followed by increased delta power in the occipital cortex. It can be speculated that the post-SD activation of the visual cortex may be perceived as positive aura symptoms.

The strength of the present study was reliable induction and recording of SD in freely behaving animals that mimics better conditions of migraine aura in patients. Further, detailed investigation of temporal evolution of cortical activity following SD is important advantage of the study. Limitations include small groups of animals that resulted from difficulties of obtaining long artifact-free ECoG recordings in freely behaving rats, and low spatial covering of SD propagation. The lack of direct electrographic evidence of SD occurrence during migraine attack in patients complicates translation of the experimental results to humans. Pathways of the non-synaptic propagation of SD over the lisencephalic cortex of rodents may differ from those in the gyrencephalic cortex of humans. Non-uniform velocity of SD propagation in gyri and sulci [44] is distinct from the constant rate of SD expansion across the lisencephalic cortex of rats. Complex spatiotemporal patterns of SD spread, including spiral and reverberating waves, seem to be more common in the gyrencephalic cortex [45].

To sum up, our study shows that slow and fast cortical oscillations exhibit pronounced difference in their vulnerability to suppressive effect of SD. In conscious drug-free brain, high-frequency gamma oscillations involved in sensory and pain processing are particularly sensitive to SD influence and show spatially broad long-lasting cessation. Why gamma activity playing the critical role in the function of the conscious brain and pain perception is more vulnerable to suppressive effects of SD in awake conditions remains unclear and needs further investigation. The state-dependent features of transient cortical dysrhythmia induced by SD should be considered in translation of experimental data to clinic of migraine and understanding pathophysiological mechanisms of migraine aura.

### Supplementary Information

**Additional file1: Fig. S1.** Photomicrograph of the typical lesion produced by the amygdala pinprick. Scale bar is 1 mm.

**Additional file 2: Fig. S2.** Effect of sham stimulation of the amygdala on ECoG power. Graphs show mean power of delta (1-4 Hz), theta (4-8 Hz), alpha (8-12 Hz), beta (12-25 Hz) and gamma (25-50 Hz) oscillations (marked on the right Y-axis) in the frontal (left fragments) and occipital (right fragments) cortices of the two hemispheres in awake rats after amygdala pinprick not triggering SD (*n*=6). Within each band, lines with shadows mark baseline activity power and circles mark power for 10-s intervals following amygdala pinprick. The sham stimulation did not change ECoG power.

**Additional file 3: Table S1.** Effects of SD on spectral power of cortical oscillations in different frequency bands in awake and anesthetized rats.

## Data Availability

The datasets used and analyzed during the current study are available from the corresponding author on reasonable request.
