# Supplementary figures and images for: Different vulnerability of fast and slow cortical oscillations to suppressive effect of spreading depolarization: state-dependent features potentially relevant to pathogenesis of migraine aura
Source: J Headache Pain. 2024 Jan 15;25(1):8. doi: 10.1186/s10194-023-01706-x (PMC10789028; doi:10.1186/s10194-023-01706-x)

**
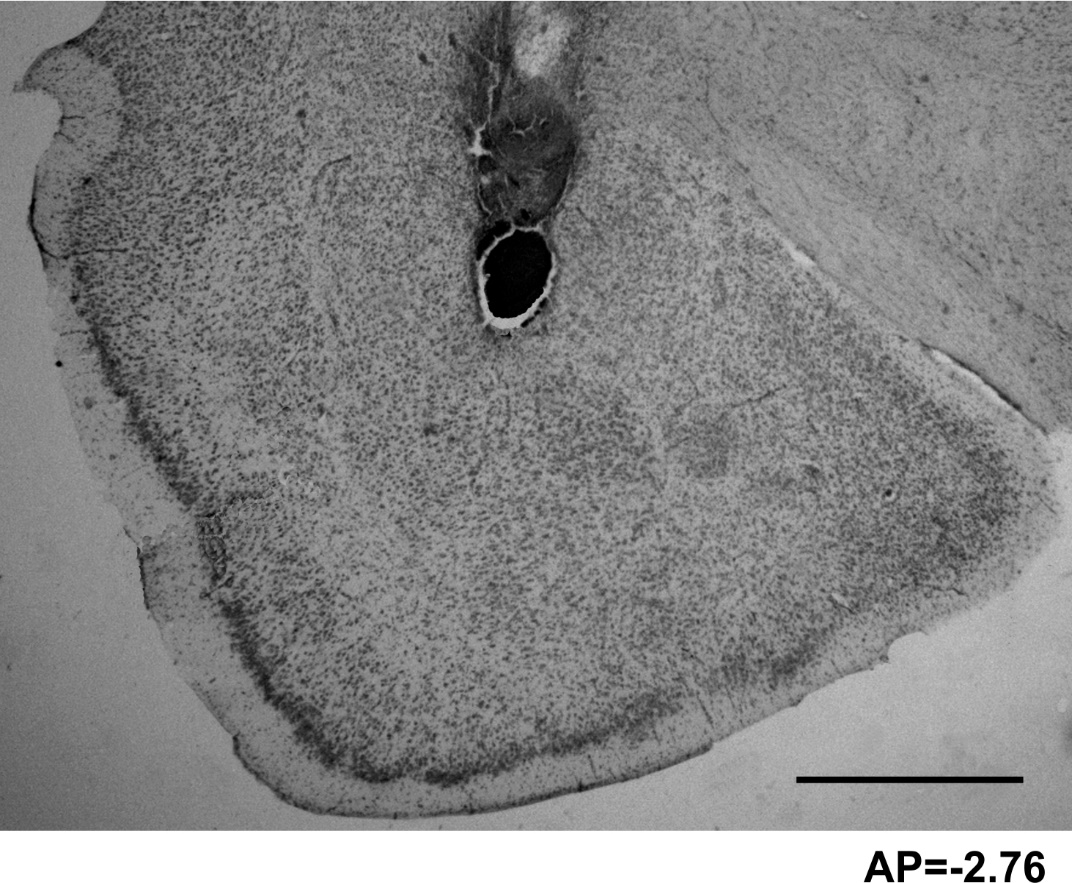
**

**Fig. S1**. Photomicrograph of the typical lesion produced by the amygdala pinprick.

Scale bar is 1 mm.

Supplement: Supplementary file 1 — Additional file1: Fig. S1. Photomicrograph of the typical lesion produced by the amygdala pinprick. Scale bar is 1 mm. [file 10194_2023_1706_MOESM1_ESM.docx]
