# Supplementary material for: Different vulnerability of fast and slow cortical oscillations to suppressive effect of spreading depolarization: state-dependent features potentially relevant to pathogenesis of migraine aura
Source: J Headache Pain. 2024 Jan 15;25(1):8. doi: 10.1186/s10194-023-01706-x (PMC10789028; doi:10.1186/s10194-023-01706-x)
Supplement: Supplementary file 2 — Additional file 2: Fig. S2. Effect of sham stimulation of the amygdala on ECoG power. Graphs show mean power of delta (1-4 Hz), theta (4-8 Hz), alpha (8-12 Hz), beta (12-25 Hz) and gamma (25-50 Hz) oscillations (marked on the right Y-axis) in the frontal (left fragments) and occipital (right fragments) cortices of the two hemispheres in awake rats after amygdala pinprick not triggering SD (n=6). Within each band, lines with shadows mark baseline activity power and circles mark power for 10-s intervals following amygdala pinprick. The sham stimulation did not change ECoG power. [file 10194_2023_1706_MOESM2_ESM.docx]

**
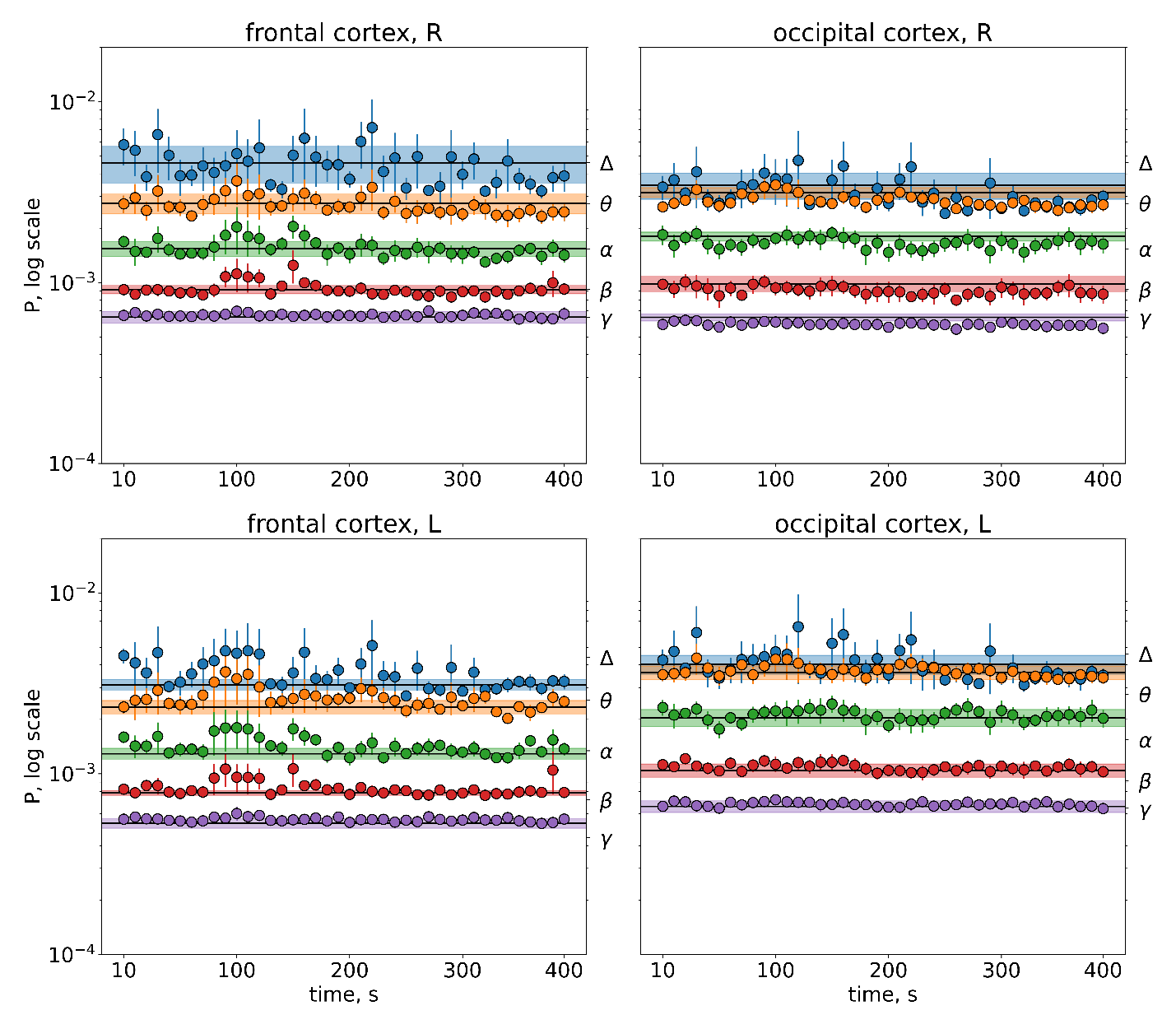
Fig. S2**. ***Effect of sham stimulation of the amygdala on ECoG power***. Graphs show mean power of delta (1-4 Hz), theta (4-8 Hz), alpha (8-12 Hz), beta (12-25 Hz) and gamma (25-50 Hz) oscillations (marked on the right Y-axis) in the frontal (left fragments) and occipital (right fragments) cortices of the two hemispheres in awake rats after amygdala pinprick not triggering SD (n=6). Within each band, lines with shadows mark baseline activity power and circles mark power for 10-s intervals following amygdala pinprick. The sham stimulation did not change ECoG power.
