# Supplementary material for: Different vulnerability of fast and slow cortical oscillations to suppressive effect of spreading depolarization: state-dependent features potentially relevant to pathogenesis of migraine aura
Source: J Headache Pain. 2024 Jan 15;25(1):8. doi: 10.1186/s10194-023-01706-x (PMC10789028; doi:10.1186/s10194-023-01706-x)
Supplement: Supplementary file 3 — Additional file 3: Table S1. Effects of SD on spectral power of cortical oscillations in different frequency bands in awake and anesthetized rats. [file 10194_2023_1706_MOESM3_ESM.docx]

**Table S1. *Effects of SD on spectral power of cortical oscillations in different frequency bands in awake and anesthetized rats***

|  | **Frontal cortex** | | | | **Occipital cortex** | | | |
| --- | --- | --- | --- | --- | --- | --- | --- | --- |
|  | ***Ipsilateral***  *(SD vs bg)* | | ***Contralateral***  *(SD vs bg)* | | ***Ipsilateral***  *(SD vs bg)* | | ***Contralateral***  *(SD vs bg)* | |
|  | F (2, 59) | p | F (2, 59) | p | F (2, 59) | p | F (2, 59) | p |
| **Awake rats** |  |  |  |  |  |  |  |  |
| Delta (1-4 Hz) | **1.916** | **<0.001** | 1,145 | 0.220 | **1,814** | **<0.001** | 0.883 | 0.719 |
| Theta (4-8 Hz) | **3.234** | **<0.001** | 1.241 | 0.112 | **2.021** | **<0.001** | 1.254 | 0.111 |
| Alpha (8-12 Hz) | **2.920** | **<0.001** | 0.890 | 0.707 | **1.369** | **<0.001** | 0.930 | 0.625 |
| Beta (12-25 Hz) | **4.133** | **<0.001** | 1.144 | 0.221 | **1.462** | **<0.001** | 0.842 | 0.794 |
| Gamma (25-50 Hz) | **9.426** | **<0.001** | **2.395** | **<0.001** | **5.701** | **<0.001** | **2.047** | **<0.001** |
| **Anesthetized rats** |  |  |  |  |  |  |  |  |
| Delta (1-4 Hz) | **5.516** | **<0.001** | 1,125 | 0.134 | **5,427** | **<0.001** | 1.171 | 0.193 |
| Theta (4-8 Hz) | **6.166** | **<0.001** | 0.990 | 0.501 | **5.225** | **<0.001** | 0.970 | 0.542 |
| Alpha (8-12 Hz) | **5.515** | **<0.001** | 1.018 | 0.444 | **4.787** | **<0.001** | 0.880 | 0.722 |
| Beta (12-25 Hz) | **5.075** | **<0.001** | 1.172 | 0.192 | **4.227** | **<0.001** | 1.226 | 0.134 |
| Gamma (25-50 Hz) | **5.949** | **<0.001** | 0.851 | 0.774 | **2.925** | **<0.001** | 1.262 | 0.109 |

Results of repeated measures ANOVA analysis of spectral power dynamics in the frontal and occipital regions of the ipsilateral and contralateral to SD hemispheres of awake (n=7) and anesthetized (n=6) rats. 600-s fragments of baseline and post-SD activity were compared within each frequency band. Time (sixty 10-s intervals) was a dependent variable; SD was an independent variable. Interaction effects between time and SD factors are shown; significant effects are marked by bold.
